# Chemical Composition and Nematicidal Activity of Essential Oil of *Agastache rugosa* against *Meloidogyne incognita*

**DOI:** 10.3390/molecules18044170

**Published:** 2013-04-09

**Authors:** He Qin Li, Qi Zhi Liu, Zhi Long Liu, Shu Shan Du, Zhi Wei Deng

**Affiliations:** 1 Department of Entomology, China Agricultural University, Haidian District, Beijing 100193, China; E-Mails: hqliaau@163.com (H.Q.L.); lqzwyz@cau.edu.cn (Q.Z.L.); 2 College of Resources Science and Technology, Beijing Normal University, Haidian District, Beijing 100875, China; 3 Analytic and Testing Center, Beijing Normal University, Haidian District, Beijing 100875, China; E-Mail: dengzw@bnu.edu.cn

**Keywords:** *Agastache rugosa*, *Meloidogyne incognita*, essential oil composition, methyleugenol, estragole, eugenol

## Abstract

The aim of this research was to determine the chemical composition and nematicidal activity of essential oil of *Agastache rugosa* flowering aerial parts against the root knot nematode, *Meloidogyne incognita*, and to isolate and identify any nematicidal constituents from the essential oil. The essential oil of *A. rugosa* aerial parts was obtained by hydrodistillation and analyzed by GC-FID and GC-MS. A total of 37 components of the essential oil were identified, with the principal compounds being methyleugenol (50.51%), estragole (8.55%), and eugenol (7.54%), followed by thymol (3.62%), pulegone (2.56%), limonene (2.49%) and caryophyllene (2.38%). Based on bioactivity-guided fractionation, the three active constituents were isolated from the essential oil and identified as methyleugenol, estragole and eugenol. The essential oil of *A. rugosa* exhibited strong nematicidal activity against *M.* *incognita*, with a LC_50_ value of 47.3 μg/mL. The components eugenol (LC_50_ = 66.6 μg/mL) and methyleugenol (LC_50_ = 89.4 μg/mL) exhibited stronger nematicidal activity against *M.* *incognita* (LC_50_ = 185.9 μg/mL). The results indicate that the essential oil of *A. rugosa* aerial parts and its constituent compounds have potential for development into natural nematicides for control of the root knot nematode.

## 1. Introduction

Nematodes are tiny worms, some of them are parasites to plants, and can play an important role in the predisposition of the host plant to invasions by secondary pathogens. *Meloidogyne incognita* (Kofoid and White) Chitwood is the most economically important and widely distributed nematode throughout China and it is responsible for considerable crop losses [1]. Essential oils from different plant sources have demonstrated several biological activities, including antibacterial and antifungal [2,3], insecticidal [4,5,6], larvicidal [7,8,9], acaricidal [10], and nematicidal [1,11,12,13,14,15,16]. As a consequence, this vast arsenal of bioactive compounds has attracted significant and increasing attention of researchers in recent years [17,18,19,20]. During our mass screening program for new agrochemicals from local wild plants and Chinese medicinal herbs, the essential oil from *Agastache rugosa* (Fisch. et Mey.) Kuntze (family: Labiatae) flowering aerial parts has been found to possess nematicidal activity towards the root knot nematode, *M. incognita*.

*A. rugosa* has been used as a wild vegetable and herbal drug for the treatment of anorexia, vomiting and other intestinal disorders [21]. Chemical composition of the essential oils obtained from aerial parts (stem, flower, leaves) of *A. rugosa* grown in different countries has been the subject of some studies and a great variation in chemical composition of the essential oils were observed [22,23,24,25,26,27,28,29,30,31,32,33,34,35,36]. This plant has been proved to have antimicrobial [26,32], anti-fungal [30,31] and antiviral activity [28]. Methanol extract of *A. rugosa* whole plant possessed strong insecticidal activity against the cigarette beetle (*Lasioderma serricorne*) [37] and adults of the rice weevil (*Sitophilus oryzae*) and the adzuki bean weevil (*Callosobruchus chinensis*) [38]. However, a literature survey has shown that there are no reports on the nematidicidal activity of *A. rugosa* essential oil against root-knot nematodes, thus we decided to investigate for the first time the chemical constituents and nematicidal activity of the essential oil of *A. rugosa* against this species and to isolate any active constituent compounds from the essential oil.

## 2. Results and Discussion

### 2.1. Essential Oil Chemical Composition

**Figure 1:**
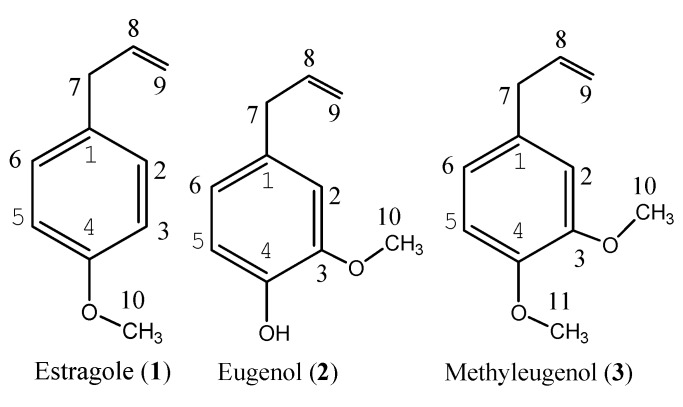
Constituent compounds isolated from the essential oil of *Agastache rugosa* aerial parts.

The yield of *A. rugosa* essential oil was 0.32% (v/w) and the density of the essential oil was determined to be 0.91 g/mL. GC-MS analysis of the essential oil of *A. rugosa* aerial parts led to the identification and quantification of a total of 37 major components, accounting for 96.14% of the total components present (Table 1). The principal compounds in the essential oil of *A. rugosa* were methyleugenol (50.51%), estragole (8.55%), and eugenol (7.54%) (Figure 1), followed by thymol (3.62%), pulegone (2.56%), limonene (2.49%) and caryophyllene (2.38%). Most of the essential oil was phenylpropanoids (66.60%) and only 14.22% monoterpenoids and 13.34% sesquiterpenoids. The results are quite different from the previous reports. For example, estragole (ranging from 46.7 to 93.7%) was major compound of the essential oils of *A. rugosa* and *A. foeniculum* and putative hybrids collected from North America [22,39]. Moreover, the essential oils of *A. rugosa* collected from Europe [23,24], Vietnam [25], and Korea [28,29,30] all contained estragole as a principal compound.

**Table 1:** Chemical constituents of the essential oil derived from *Agastache rugosa* aerial parts.

RI *	Compound	Composition, %
Monoterpenoids		14.22
931	α-Pinene	0.52
984	β-Pinene	1.21
1029	D-Limonene	2.49
1057	γ-Terpinene	0.13
1188	α-Terpineol	0.34
1097	Linalool	1.77
1236	Pulegone	2.56
1288	Cuminic alcohol	1.38
1292	Thymol	3.62
Sesquiterpenoids		13.34
1313	Elixene	0.12
1317	Carvacrol	0.20
1350	α-Cubebene	0.13
1382	*iso*-Ledene	0.11
1385	β-Bourbonene	0.37
1393	β-Elemen	0.26
1420	Caryophyllene	2.38
1462	*cis*-α-Farnesene	0.17
1473	γ-Muurolene	0.41
1486	Germacrene D	1.45
1491	Aromadendrene	0.32
1499	Bicyclogermacrene	0.88
1500	α-Muurolene	0.29
1511	α-Farnesene	0.72
1454	α-Caryophyllene	0.19
1521	δ-Cadinene	0.85
1546	Cadina-4,9-diene	0.28
1561	Germacrene B	0.68
1578	Spatulenol	1.11
1584	Caryophyllene oxide	0.73
1592	Viridiflorol	0.12
1642	τ-Muurolol	0.81
1652	α-Cadinol	0.96
Phenylpropanoids		66.60
1195	Estragole	8.55
1356	Eugenol	7.54
1369	Methyleugenol	50.51
Others		1.98
975	Morrilol	0.16
1066	Acetophenone	1.82
Total identified		96.14

* RI, retention index as determined on a HP-5MS column using the homologous series of *n*-hydrocarbons.

On the other hand, thymoquinone (70.11%) was the main component of the essential oil of *A. rugosa* aerial parts collected from northeast China [40], while estragole (74.84%) was the major compound of the essential oil of *A. rugosa* aerial parts collected from Hubei Province (Central China) and methyleugenol (49.89%) and estragole (19.45%) were two major compounds of the essential oil of *A. rugosa* aerial parts collected from Henan Province (Central China) [41]. Pulegone (37.58%) was the principal component of the essential oil of *A. rugosa* aerial parts collected from Zhejiang Province (Eastern China) [42]. Three chemoecological types of *A. rugosa* were consequently suggested as follows: type A with estragole as the main constituent, type B with methyleugenol as the principal constituent and type C with menthone derivates as the major constituents [43]. It seems that all the three chemotypic races of this species of plant are found in China. Our samples (from Beijing) belong to type B, with about 50% methyleugenol as the principal compound (Table 1), whereas samples collected from Central China belong to type A or type B [41]. Moreover, samples collected from Eastern China and Northeast China do not belong to any of the three types [40,42] because the major constituent compounds in those essential oils were thymoquinone and pulegone, respectively. Thus, further studies are needed to clarify variation of constituents of the Chinese *A. rugosa* oil. However, all these differences of chemical composition of the essential oils might have been due to harvest time and local, climatic and seasonal factors, as well as storage duration of the medicinal herbs. For practical use, it would be necessary to standardize the essential oil of Chinese *A. rugosa*.

### 2.2. Nematicidal Activity

The essential oil of *A. rugosa* aerial parts exhibited strong nematicidal activity against root knot nematode, *M. incognita*, with an LC_50_ value of 47.3 µg/mL (Table 2). Compared with the synthetic insecticide carbofuran, the essential oil of *A. rugosa* aerial parts possessed stronger toxicity against *M. incognita*, as carbofuran displayed a LC_50_ value of 72.3 μg/mL [1]. Among the three main components, both eugenol (LC_50_ = 66.6 μg/mL) and methyleugenol (LC_50_ = 89.4 μg/mL) exhibited stronger nematicidal activity against *M. incognita* than estragole (LC_50_ = 185.9 μg/mL). Compared with carbofuran, eugenol and methyleugenol exhibited a stronger or similar level of nematicidal activity towards *M. incognita*. In previous reports [44,45,46,47], eugenol and methyleugenol have been demonstrated to possess nematicidal activity against several nematode species, e.g., pine wood nematode (*Bursaphelenchus xylophilus*), *Caenorhabditis elegans*, and root knot nematode, *M. incognita*. However, all the three isolated constituent compounds possessed less activity against the root knot nematode than the crude essential oil (Table 2), suggesting that there may be stronger active compound(s) in small amounts in the essential oil or maybe some synergistic action between the various compounds. For example, carvacrol and thymol immobilized the juveniles of the root knot nematode *M. javanica* and inhibited hatching at >125 μL/L *in vitro* and the two components mixed in sandy soil at concentrations of 75 and 150 mg/kg reduced root galling of cucumber seedlings [17]. Carvacrol and thymol exhibited nematicidal activity against root knot nematode, *M. incognita*, with 76 h LC_50_ values of 176 μg/mL and 280 μg/mL, respectively [45]. In previous reports, a synergistic effect among terpene constituents of the essential oils from seven plants indigenous to Greece has been detected [18,46]. The most potent terpene pairs between which synergistic actions were found, in decreasing order, were: *trans*-anethole/geraniol, *trans*-anethole/eugenol, carvacrol/eugenol and geraniol/carvacrol.

**Table 2:** Nematicidal activity of the essential oil of *Agastache rugosa* aerial parts and its three main components against *Meloidogyne incognita*.

Treatments	Concentrations (μg/mL)	LC_50_ (μg/mL) 95% FL *	LC_90_ (μg/mL) 95% FL *	Slope ± SE	Chi square (χ^2^ )
*A. rugosa*	12.5-200.0	47.3 (42.9–55.2)	174.6 (156.9–191.1)	6.51 ± 0.66	9.06
Estragole	80.0-860.0	185.9 (169.7–206.1)	463.6 (422.9–489.5)	7.03 ± 0.68	7.64
Eugenol	12.5-200.0	66.6 (60.6–74.1)	182.3 (164.8–198.6)	5.21 ± 0.53	9.77
Methyleugenol	40.0-240.0	89.4 (79.7–98.1)	193.7 (176.7–214.9)	8.36 ± 0.78	8.25
Carbofuran **	25.0-400.0	72.3 (37.9–118.0)	-	-	13.57

* Fiducial limits; ** From Bai *et al.* [11].

Andres *et al.* [18] also demonstrated that there was a synergistic interaction between some of the constituent compounds (carvone, 1,8-cineole and menthol) at certain concentrations. Considering the positive control is a synthetic insecticide, the observed nematicidal activity of the essential oil of *A. rugosa* and the three constituent compounds, especially eugenol and methyleugenol, is quite promising and the essential oil and its individual constituents show potential to be developed as possible natural nematicides for the control of the root knot nematodes, although for the practical application of the essential oil and the isolated constituent compounds as novel nematicides, further studies on the safety of the essential oil/pure compounds to humans and on development of formulations are necessary to improve the efficacy and stability and to reduce cost.

## 3. Experimental

### 3.1. Plant Material and Essential Oil Extraction

Fresh aerial parts (10 kg of leaves, stems and flowers) of *A. rugosa* were harvested in August 2009 from Xiaolongmeng National Forest Park (Mentougou District, Beijing 102300, 38.24° N latitude and 115.20° E longitude). The species was identified by Dr. Liu, QR (College of Life Sciences, Beijing Normal University, China), and the voucher specimen (CMH-TuHuoXiang-Beijing-2009-08) was deposited in the museum of Department of Entomology, China Agricultural University. The aerial parts were air-dried for one week and ground to a powder using a grinding mill (Retsch Muhle, Germany). The powder was subjected to hydrodistillation using a modified Clevenger-type apparatus for 6 h and extracted with *n*-hexane. Anhydrous sodium sulphate was used to remove water after extraction. The essential oil was stored in airtight containers in a refrigerator at 4 °C for subsequent experiments. 

### 3.2. Gas Chromatography-Mass Spectrometry

Components of the essential oil of *A. rugosa* aerial parts were separated and identified by gas chromatography-flame ionization detection (GC-FID) and gas chromatography-mass spectrometry (GC-MS) using an Agilent 6890N gas chromatography system connected to an Agilent 5973N mass selective detector. The same column and analysis conditions were used for both GC-FID and GC-MS. They were equipped with capillary column with HP-5MS (30 m × 0.25 mm × 0.25 μm). The GC settings were as follows: the initial oven temperature was held at 60 °C for 1 min and ramped at 10 °C min^−1^ to 180 °C where it was held for 1 min, and then ramped at 20 °C min^–1^ to 280 °C and held there for 15 min. The injector temperature was maintained at 270 °C. The samples (1 μL, after diluted to 1% with acetone) were injected, with a split ratio of 1: 10. The carrier gas was helium at flow rate of 1.0 mL min^–1^. Spectra were scanned from 20 to 550 *m/z* at 2 scans s^–1^. Most constituents were identified by gas chromatography by comparison of their retention indices with those of the literature or with those of authentic compounds available in our laboratories. The retention indices were determined in relation to a homologous series of *n*-alkanes (C_8_–C_24_) under the same operating conditions. Further identification was made by comparison of their mass spectra with those stored in NIST 05 and Wiley 275 libraries or with mass spectra from the literature [48]. Relative percentages of the individual components of the essential oil were obtained by averaging the GC-FID peak area% reports.

### 3.3. Purification and Characterization of Three Constituent Compounds

The crude essential oil of *A. rugosa* aerial parts (25 mL) was chromatographed on a silica gel (Merck 9385, 1,000 g) column (85 mm i.d., 850 mm length) by gradient elution with a mixture of solvents (*n*-hexane, *n*-hexane-ethyl acetate). Fractions of 500 mL were collected and concentrated at 40 °C, and similar fractions according to their TLC profiles were combined to yield 15 fractions. Fractions 5–8, 10, 12 that possessed nematicidal toxicity, and with similar TLC profiles, were pooled and further purified by preparative silica gel column chromatography (PTLC) with petroleum ether-acetone (50:1, v/v) until to obtain the pure compounds for determining their structures based on nuclear magnetic resonance spectroscopy. ^1^H and ^13^C-NMR spectra were recorded on a Bruker AMX500 [500 MHz (1H)] instrument using CDCl_3_ as the solvent with TMS as internal standard. Electron impact mass spectra (EIMS) were determined on a Micromass VG7035 mass spectrometer at 70 eV (probe).

### 3.4. Isolated Constituent Compounds

*Estragole* (**1**, Figure 1). A colorless oil (0.3 g), C_10_H_12_O. ^1^H-NMR (CDCl_3_) δ (ppm): 7.27 (1H, d, H-3, 5), 7.01 (1H, d, H-2, 6), 6.14 (1H, m, H-8), 5.26 (2H, d, H-9), 3.92 (3H, s, 10-CH_3_), 3.50 (2H, d, H-7). ^13^C-NMR (125 MHz, CDCl_3_) δ (ppm): 158.22 (C-8), 138.08 (C-1), 132.16 (C-9), 129.61 (C-4), 115.52 (C-3, C-5), 113.99 (C-2, C-6), 55.18(C-10), 39.52 (C-7). EI-MS *m/z* (%): 149 (13), 148 (100), 147 (46), 133 (23), 121 (41), 117 (32), 105 (23), 91 (20), 77 (23). The spectral data matched that given in a previous report [6].

*Eugenol* (**2**, Figure 1). A colorless oil (0.4 g), C_10_H_12_O_2_. ^1^H-NMR (CDCl_3_) δ (ppm): δ: 6.83 (1H, d, *J* = 8.8 Hz, H-5), 6.66 (1H, dd, *J* = 8.4, 1.8 Hz, H-6), 6.65 (1H, d, *J* = 1.8 Hz, H-2), 5.94 (1H, m, H-8), 5.73 (1H, br.s, D_2_O exchangeable, -OH), 5.06 (2H, m, H-9), 3.81 (3H, s, -OCH_3_), 3.30 (2H, dt, *J* = 6.6, 1.5 Hz, H-7). ^13^C-NMR (125 MHz, CDCl_3_) δ (ppm): 146.60 (C-3), 144.03 (C-4), 137.91 (C-8), 131.94 (C-1), 115.49 (C-5), 114.46 (C-2), 111.26 (C-9), 55.84 (C-10), 39.92 (C-7). EI-MS *m/z* (%): 165 (11), 164 (100), 149 (29), 137 (15), 133 (15), 121 (14), 103 (19), 91 (14), 77 (21), 55 (18). The spectral data matched that given in a previous report [49].

*Methyleugenol* (**3**, Figure 1). A colorless oil (0.8 g), C_1__1_H_1__4_O_2_. ^1^H-NMR (CDCl_3_) δ (ppm): 6.81 (1H, d, *J* = 8.0, H-6), 6.74 (1H, dd, *J* = 8.0, 2.0, H-5), 6.71 (1H, d, *J* = 2.0 H-2), 5.96 (1H, ddt, *J* = 17.0, 10.4, 6.8, H-8), 5.07 (2H, m, H-9), 3.86 (3H, s, -OCH_3_), 3.87 (3H, s, -OCH_3_), 3.34 (2H, ddd, *J* = 6.8, 1.7, 1.4, H-7). ^13^C-NMR (125 MHz, CDCl_3_) δ (ppm): 148.8 (C-4), 147.3 (C-3), 137.7 (C-8), 132.6 (C-6), 120.4 (C-1), 115.6 (C-9), 111.8 (C-2), 111.2 (C-5), 55.9(-OCH_3_), 55.7 (-OCH_3_), 39.8 (C-7). EI-MS *m/z* (%): 178 (100), 163 (33), 151 (15), 147 (32), 135 (18), 131 (11), 107 (28), 103 (39), 91 (50), 77 (26). The spectra data matched with previous report [50].

### 3.5. Nematicidal Assay

Egg masses of *M. incognita* obtained from tomato roots with aid of a stereomicroscope were maintained in Petri dishes during 24 h in distilled H_2_O for the juveniles to hatch. Range-finding studies were run to determine the appropriate testing concentrations. A serial dilution of *A. rugosa* essential oil (five concentrations) and pure compounds (five concentrations) was prepared in H_2_O solution with 2% DMSO. Aliquots of H_2_O (20 μL) containing ca. 100 juveniles (J_2_) were transferred to vials to which 980 μL of the solution containing essential oil or pure compounds was added. The vials were kept in a hood at 25 °C. The counting of the inactive nematodes was performed at every 24 h for 72 h. After the last count, the inactive juveniles were maintained in distilled H_2_O for 24 h to observe their revival. Six repetitions for each treatment were performed using H_2_O and a 2% DMSO in H_2_O solution as control. The experiments were repeated in three times. Results from all replicates for the pure compounds and essential oil were subjected to probit analysis using the PriProbit Program V1.6.3 to determine LC_50_ (median lethal concentration) values and their 95% confidence intervals (CI 95%) [51].

## 4. Conclusions

The study indicates that the essential oil of *A. rugosa* aerial parts and its main constituent compounds, eugenol, methyleugenol and estragole, have potential for development into natural nematicides for the control of root knot nematodes.
